# Fictitious cases as a methodology to discuss sensitive health topics in focus groups

**DOI:** 10.1080/17482631.2023.2233253

**Published:** 2023-07-18

**Authors:** Emma Grundtvig Gram, John Brandt Brodersen, Cæcilie Hansen, Kristen Pickles, Jenna Smith, Alexandra Ryborg Brandt Jønsson

**Affiliations:** a The Research Unit for General Practice and Section of General Practice, Department of Public Health, University of Copenhagen, Copenhagen, Denmark; b Primary Health Care Research Unit, Region Zealand, Denmark; c The Research Unit for General Practice, Department of Social Medicine, University of Tromsø, Tromsoe, Norway; d Faculty of Medicine and Health, The University of Sydney, Sydney, Australia; e Department of People and Technology, Roskilde University, Roskilde, Denmark

**Keywords:** PSA screening, prostate cancer, focus groups, sensitive topics, research methodology, health communication, cancer, cancer screening, projective techniques

## Abstract

**Purpose:**

It can be challenging to research aspects of people’s health behaviour, attitudes, and emotions due to the sensitive nature of these topics. We aimed to develop a novel methodology for discussing sensitive health topics, and explore the effectiveness in focus groups using prostate cancer and screening as an example.

**Method:**

We developed a fictitious case and employed it as a projective technique in focus groups on prostate cancer and screening. The participants were men and their partners who lived in Denmark.

**Results:**

The technique encouraged emotional and cognitive openness in focus group discussions about the risk of prostate cancer, the benefits and harms of screening, and decision-making about screening. It appeared that using the fictitious case allowed the participants to personally distance themselves from the topic, project emotions onto the case, and thereby openly talk about their emotions.

**Conclusion:**

This article presents a methodological contribution to communication about sensitive topics in focus groups, using prostate cancer screening as an example. Further refinement of the methodology is needed to enable participants to transfer improvements in knowledge to their own decision about screening.

## Introduction

Cancer and cancer screening involve private domains of people’s lives. They can be considered sensitive fields of inquiry, so the methods that can be applied to study them are limited (Birenbaum‐Carmeli et al., 2008). Traditional focus groups are intuitively not appropriate to elicit people’s views on sensitive topics and private spheres (Farquhar, 1999; Pennebaker et al., 1987). Yet, several creative approaches have been developed to facilitate research of such personal aspects of subjects’ lives (Birenbaum‐Carmeli et al., 2008). This has allowed the focus-group format to become accepted as a method to explore sensitive topics (Brondani et al., 2008; de Oliveira, 2011; Wellings et al., 2000). One method to supplement focus groups on sensitive topics is called “projective techniques”, whereby the subjects project their feelings onto a media and indirectly into their responses. Many of such approaches are based on protreptic principles of self-reflection, motivation, and non-judgemental spaces (Kirkeby, 2016; Marshall, 2020). Media include pictures, word associations, jokes, books, imaginary narratives, vignettes, painting, and tasting (Brittany, 2020; Brondani et al., 2008; Richman, 1996; Rook, 1988). This process provides valuable insight into how subjects feel about a sensitive topic that they otherwise may not have disclosed. Overall, projective techniques can allow for feelings about sensitive topics to be communicated effectively, openly, and honestly, and can provide greater depth to researchers’ understanding of how subjects feel about sensitive topics.

In market research, projective techniques are used to identify consumers’ preferences and values (Esmerino et al., 2017; Pettigrew & Charters, 2008). Market research suggests that the use of thought experiments as a strategy in dialogues make people reveal something about themselves when they translate the general into something specific (Kirkeby, 2016; Marshall, 2020). Likewise, educational researchers have used thought experiments as projective techniques and argue that it allows individuals to insert the narrative into the context of their own experience and thereby reveal familiar conditions, norms, conventional practices, and values that these rely upon (Barone, 2001; Watson, 2011). Health research may gain from adopting such projective techniques (Shaffer et al., 2018).

One example of a sensitive topic in health research is men’s emotional and cognitive response to information about the benefits and harms of prostate cancer screening (Nielsen et al., 2020). Worldwide, prostate cancer is the third most common cancer diagnosed and the fifth leading cause of mortality among men (Sung et al., 2021). Incidence rates vary substantially across the world but are closely associated with the clinical use of the prostate-specific antigen (PSA) screening test (Potosky et al., 1995). Internationally, there is no population-based screening program for prostate cancer due to concerns about high rates of overdiagnosis and overtreatment (Carter et al., 2015; Glasziou et al., 2020; Moyer, 2012). However, the PSA test is frequently used by doctors in many countries to opportunistically screen asymptomatic men for prostate cancer risk, including Denmark and Australia although this is not recommended by national guidelines.

In a Danish interview study with men potentially overdiagnosed with prostate cancer, the researchers had difficulties getting the participants to talk about how the diagnosis affected their lives emotionally and socially. In the interviews, the men were asked to complete the Consequences Of Screening questionnaire, which encouraged the men to continue the interview (Brodersen et al., 2010; Nielsen et al., 2020). In other words, the questionnaire served as a projective technique through which feelings and experiences were more easily expressed.

To this end, we developed a fictitious case as a projective technique to allow projection and distancing from the sensitive nature of prostate cancer and PSA testing while encouraging emotional and cognitive openness. We aimed to explore the usefulness of this methodology in focus groups using prostate cancer and PSA screening as an example.

## Navigating health behaviour

Matters of health behaviour are present in most peoples’ everyday life. This is evident in navigating and negotiating treatments for medical conditions, dietary and physical activity choices, symptom awareness, and other healthcare-seeking activities such as participating in medical screening programs. Citizens are expected to actively engage in maintaining their physical and mental health, and thus society has come to rely heavily on personal responsibility (Foucault, 1991). Ironically, these matters come to the fore when individuals, for example, are smoking or not regularly attending a doctor, which in public discourses are viewed as a failed attempt to exert sufficient control over health behaviour (Crawford, 1980; Kristensen et al., 2016). This causes a divide in society in which moral modalities categorize individuals and individuals who do not fulfill the perceived deeds of a good citizen will come to be regarded as ignorant (Jønsson et al., 2020). Thus, understanding health behaviour among citizens should encompass social, cultural, and moral forces. This means that in order to discuss a sensitive topic that could be regarded as controversial or amoral (e.g., a poor health behaviour), a respectful and confidential space needs to be established. While many scholars have succeeded in doing this on an individual basis through ethnographic studies, investigating negotiations in group discussions requires novel approaches. It has been established that individuals are capable of discussing sensitive topics if they are relatable but not directly associated to themselves (Karimi et al., 2019). While focus groups are not intuitively appropriate to explore sensitive topics, what we refer to here as a “fictitious case” employed as a projective technique might offer participants safety to address sensitive topics in situations where discussions are clearly regulated.

## Methods

To explore if a case employed as a projective techniques could enhance discussions about prostate cancer and PSA testing, we created a fictitious case and tested it in interactive focus groups. The case should offer participants safety from the sensitive nature of prostate cancer and PSA testing, and the potential shield from the embarrassment that can arise in dialogue about sensitive topics (Farquhar, 1999). We employed the ficticious case as a projective technique and therefore will refer to that as the projective technique.

In addition to the projective technique, we employed three elements that we considered important for successful conduct: collective tasks, minimal-moderator intervention, and homogenous group composition.

*Collective tasks*: We utilized collective tasks to promote group interaction based on the assumption that solving a task together would incite participants to negotiate solutions. The tasks were based on the fictitious case to encourage the participants to adopt the role of the people presented in the case and simulate events and thoughts through their eyes; so-called experience-taking (Kaufman & Libby, 2012). By these means, the tasks should encourage participants to reveal their values and heuristics, and to explain and defend any dissimilar within-group perspectives (Barbour & Kitzinger, 1999; Halkier, 2015; Hansen, 2004; Kitzinger, 1990). Participants will have to make moral or value-based judgements that may reveal what shapes their perspective.

*Minimal-moderator intervention*: We assumed that the researcher who moderates the focus groups would be seen as holding authority over the appropriateness of answers related to prostate cancer and PSA testing offered by participants (Halkier, 2010; Puchta & Potter, 2002). Within the topic of prostate cancer, health behaviour, and testing, we assumed that norms about appropriateness would be especially pronounced compared to other fields. To avoid moderator bias and to adhere to the fact that interaction is a large and relevant part of forming opinions, the focus groups were left without a moderator during the collective tasks (Puchta & Potter, 2002). Further, we assumed that the tasks would encourage participants to focus on one another rather than the moderator.

*Homogenous group composition*: The group composition is important in focus group research (Farquhar, 1999). We prioritized ensuring that the groups were relatively homogenous so that participants would be more inclined to feel cohesion and be open to discussing prostate cancer and PSA testing with their peers (Owen, 2001). We assumed that the more cohesion within the group, the less of an impact the researcher would have as an authority (Farquhar, 1999). How and to what extent discussion is regulated depends on the context, and we assumed that discussion would be less regulated if groups were sex stratified and relatively homogeneous.

### Study population

We directed the invitation for participation to people with a prostate, hereby referred to as “men”. We encouraged the men to involve their partners, as we know from previous research that partners play a role in health literacy especially among men and in relation to prostate cancer screening (Nielsen et al., 2020). We approached men who were above the age of 55 years through advertising on social media and through the snowball-method (Naderifar et al., 2017). There was no upper age limit and no requirements to have had PSA testing done before. The only exclusion criterion was a previous diagnosis of prostate cancer. There were no exclusion criteria for partners’ sex, yet all partners presented as female.

### Conduct of focus groups

We simultaneously conducted one focus group with men and one focus group with their partners. To begin, the two groups were assembled in one room to receive information about prostate cancer and PSA testing. The information consisted of an oral presentation and three videos that were official governmental information pamphlets and videos for lay people (The Danish Cancer Society, 2022; Midtjylland, 2015). The material communicated the dilemma of opportunistic prostate cancer screening and covered the disease of prostate cancer, and the potential benefits, harms, and uncertainty of the PSA test, e.g., overdiagnosis and overtreatment (Glasziou et al., 2020; Nielsen et al., 2020; Ostero & Brodersen, 2018; Pathirana et al., 2022; Welch & Black, 2010).

### The case

The fictitious case was developed in a brainstorming session between authors and was based on the theory presented above, and experiences from prior knowledge, clinical work and contact with general practice in Denmark.

The Case:

The narrative began with a middle aged man named Flemming going to see his doctor to get a PSA test. He did not have any symptoms, he just wanted to get it done to confirm that nothing was wrong. He knows that the risk of having prostate cancer increases with age and that it is widespread. He is often reminded about the risk of cancer through media, governmental and non-governmental campaigns, and he and his wife Anne thought it might be a good idea to get tested.

The participants were asked to imagine that Flemming receives the same information that they just did (the governmental pamphlet-material) from his doctor while being told that Flemming’s doctor will not perform a PSA on him as he is asymptomatic. Flemming is sent home without a test, and that’s where the case was left for now.

### Implementation of the strategy

After the delivery of information, all participants were introduced to the case. Hereafter, the male participants were separated from their partners making the groups stratified by risk of prostate cancer as all the partners presented as female.

The participants of both focus groups were first asked to hierarchically sort different sections of information about prostate cancer and PSA testing (Kitzinger, 1990), according to what they believed that Flemming and Anne would find most important to decide whether or not to have a PSA test. The sections of information were worded identically to the information presented, and were written on pieces of paper which allowed the participants to arrange the information physically in front of them and perform the task as a group activity. The participants were asked to agree on the hierarchy of information, which made the activity deliberative. While this activity took place, the moderator left the room. When the moderator came back, she asked what they had found out or talked about while she was gone.

In the second part, the participants were instructed to construct a timeline of Flemming’s course of action after he returned home from the doctor’s office. Participants were asked to write notes of actions and stick them to the timeline. This activity also encouraged deliberation as the participants could only do one timeline and therefore had to reach an agreement regarding what should be put onto the timeline. The moderator again left the room and participants were again asked to present their ideas when the moderator came back.

### Analysis strategy

The focus groups were video recorded and observations were noted immediately after. Recordings were transcribed verbatim and transcriptions were coded in NVivo version 12. We chose to video record the focus groups to consider non-verbal group interaction and non-verbal emphasis of statements in the analysis.

We coded the focus groups iteratively and refined codings as new themes emerged. First coding was done inductively with no predetermined themes as guidance for the evaluation of the method. However, we focused on how the participants interacted with the case and the applied methods and how that interaction affected understanding and contextualization of knowledge and ability to express emotions. As a part of the iterative process, refining the codes, we focused on what values were associated to the statements and explanations of concepts, for example, what values did the participants tie to concepts of false negatives or overdiagnosis. We also focused on the use of pronouns; when statements were directed at the case and when they were personal or indefinite. We systematically searched transcripts for the use of all pronouns and recorded their frequency and in what situations these were used. The analysis also explored the nature of the participants’ posed arguments.

### Research ethics

The Danish Research Ethical System does not require ethical approval of qualitative studies. Personal identifiers, such as names, were altered to secure anonymity. The participants signed a form, where they were informed about their rights as informants and we received written informed consent to video record the focus groups and report findings anonymous. The participants informed us that they had appreciated participating in the focus groups and expressed gratitude for the gained knowledge.

## Results

Eight participants distributed across two focus groups participated in the study; four men and their female partners. In the following, we present results providing preliminary support that the projective methodology allowed the participants to: 1) establish common grounds and 2) justify opinions. Further, we will argue that the fictitious case functioned 3) as a shield from embarrassment, and 4) was a means to display emotion and 5) process information.

### Establishing common grounds

The use of the pronoun *we* was used to establish common ground within the groups. The use of *we* revealed what the groups considered as rational medical-consumer behaviour and which values these relied upon. The groups based their opinions about screening on financial costs associated with PSA screening. For example, many participants argued pro screening relying on the argument that a blood sample is not expensive, many blood samples are done everyday, and that drawing blood is not an advanced technology.

The partners also argued pro screening to minimize the likelihood of losing their husbands to prostate cancer, and thereby revealed that screening decisions also rely on values associated to love and relationships. In a conversation about the severity of prostate cancer, Hanne emphasized that everyone knows someone to has prostate cancer or someone who has died from it. Helle supported this and said that:

“We have reached a certain age, where these things (disease and death) come to be more and more relevant … ”. (Helle, partner)

On this ground, all the women argued that they would make their husbands call their GP and get the PSA test done. One example was Susanne:

Yeah, so if I was Anne, then I would probably make him book a new appointment with his doctor to get the test and tell Flemming: ‘Now, you will have to go!’ … if it is cancer and nothing is done then he will become ill and at one point … (pause) One has to go to the doctor quick. (Susanne, partner)

### The case as a way to justify opinions

The participants were able to communicate their desires, opinions, and worries under the guise of the case. By this, they avoided displaying opinions as their own while they still managed to communicate effectively. For example, the participants generally inferred that they thought Flemming should consult his doctor again and demand PSA testing, and justified their statements with claims of Flemming being “the worried type”:

I think that if he is the worried type and he gets this information, then I think he would want to go see his doctor again (Helle, partner)

But I also wonder: How worried is he? Well, if you are the worried type, then Anne and Flemming does not necessarily talk about this very much, and then she would probably have him book another doctor appointment in a week and tell him: You must go now, okay? (Susanne, partner)

The male participants also called the couple out as worried types. One participant (Niels), stated that Flemming should have the test done, because it was most probable that the test was negative and he, therefore, would be relieved and feel reassured. This was supported by the rest of the male group. We interpret that the use of ’the worried’ stereotype justified opinions of personal desire to get tested despite having received information about the harms of screening.

### The case as a shield from embarrassment and prejudice

In the below quote, Jens distanced the male group from the statements about Flemming by using the pronoun *we* and engaging the group in the argument:

So we assume that Flemming must be the worried type, because he, contrarily from what we would do, go to the doctor without any symptoms, to get tested for something he has no symptoms of. Goddamn, Flemming, we don’t get you at all. (Jens)

Jens spoke on behalf of the group and established rational medical-consumer behaviour in the group as opposed to the irrationality that Flemming would want to get tested. Thereby, Jens also shielded himself from the embarrassment or jugdement that could be associated with breaking from the group norm about rational medical-consumer behaviour by saying that Flemming would want to get tested. Hereby, Jens used the case to imagine the experiences of Flemming and distanced himself from that.

In other situations, the participants distanced themselves from the case by using indefinite pronouns such as *You*, *they*, and *somebody*. Participants also accompanied statements about Flemming and Anne by humour and sarcasm which further served to distance themselves from the “worried type”.

### The case as a means to display emotions

In the male group, the participants talked about what Flemming would do after the doctor declined PSA testing:

“Well, it might be that he is still worried … Okay, but what can he then do? Either he would sit down and think: well, I am really not okay with this. Would he then go back to his doctor and say: ‘Doctor, I am still worried’ or what would he do? Maybe he joins a Facebook group or. hmm, what do I know. Then he would get bombarded with things that can go wrong and then what? I don’t know, but I think he would go to see his doctor again” (Ole)

It appeared that Ole related to Flemming’s situations and imagined what he himself would feel and do. He might intuitively know that this action could be considered as overreacting but was able to express these emotions through Flemming’s case. Niels immediately followed to re-establish rational behaviour:

“But then he would go see his doctor again. The doctor would probably say: ‘Flemming, you are overreacting, let go of it’. And then Flemming would find a private provider and have the test done. And then the doctor would just be another stupid doctor.” (Niels)

These two quotes showed how the case was used to display emotions. The use of Flemming revealed emotions related to the case and how the participants imagined reacting from these emotions, for example, by seeking private testing to reduce worry. The participants all suggested that Flemming most likely would search the internet for prostate cancer and PSA testing and suddenly feel all the symptoms, which they imagine would cause him further worries.

### The case as a means to process information

Both men and their partners repeated statements about false positives, false negatives, overdiagnosis, and unnecessary treatment correctly. Correctly interpreted information was mostly expressed in terms of Flemming’s situation, for example, the following participant demonstrates accurate understanding of false negative and overtreatment:

Okay, if we perform the test, then it is not always that it (the cancer) will be detected. Even though we perform the test and it shows that he (Flemming) has a low PSA, then it is not certain that he does not have prostate cancer. And even though we perform the test and it is not positive, he can still have cancer. If he is going into treatment, we will have to treat 27 while only one will need it, because we don’t know if it’s dangerous or not. (Niels)

The men also used Flemming to reflect about the harms of screening in relation to themselves:

”Well, why not have the test done. I feel him on this one. Because you can say: if he indeed has high PSA, then okay … should he go into treatment? I am not sure that I would do it, unless something specific had happened” (Jens)

Again, when the group discussed the potential harm, they used the pronoun *we* to talk about what they, as a group, would do:

But I am thinking: why should we take this test?… We talked about the fact that we are all those types, or at least we like to think that we are, those types that just would like to know. You know, we would like to know whether we can consider yourselves as exonerated or not, or almost … (Ole)

This quote shows that the group format allowed the *we*-state of taking. The use of *we* as pronoun served to distance the participants to the sensitivity of making a decision about getting tested or not. Further, it made the participants able to relate the information to their own decision of testing. However, despite this, the participants overemphasized the benefits of PSA screening and interpreted numbers so they were in favour of screening:

… But if the test is somehow reliable, then I would get that test done. And if I was among the 773 (who would have a negative PSA test) then I would be relieved and say: Puhh! That was nice. And then there might be a small chance that the result was false. (Jens)

Jens referred to the test as “somehow reliable” after having demonstrated understanding of the fact that it is not reliable and then assessed the probability of a false result as “small”. The group of men further discussed the practicalities of the PSA test and Henrik added:

“But all of us have had a blood test done before - that happens regularly. You go to see your doctor and have a blood sample drawn and then it is not that bad and it gets examined and if you are in the big group (with a negative test result), you know that you are ‘home free’ … That would be nice.” (Henrik)

Henrik had previously shown understanding of false results, and therefore he would also know that a negative result does not equal no cancer. Niels continued immediately after:

Yes, the odds are good. To go and get some peace of mind. I would, therefore, like to have the test done (Niels)

Despite the fact that all participants understood the different types of harms of PSA screening, they all downplayed them and overemphasized arguments that supported the decision to get tested.

## Discussion

We found that the projective methodology allowed participants in this study to feel safe in sharing their opinions about PSA testing for prostate cancer. Based on this result, we argue that the fictitious case gave the participants the opportunity to justify opinions while avoiding embarrassment and judgement when opinions were contradictory to rational medical-consumer behaviour. The case served as a medium through which the participants could understand benefits and harms of screening and relate this to their own situation. However, participants found it difficult to transfer this improvement in knowledge to their own PSA testing decision.

Generally, lay people are increasingly becoming more informed of medical terms, conditions, and treatments, which improves comprehension of the potential benefits and harms of screening. However, research has found that information about the different types of harms can be difficult for individuals to relate to their own decision about screening (Jensen et al., 2021). Lay people oppositely express great enthusiasm for cancer screening (Degeling et al., 2018; Scherer et al., 2019; Schwartz et al., 2004; Waller et al., 2015), and when making the decision to participate in screening, people might not evaluate the benefits and the harms equally (Festinger, 1957; Hodson, 2020). This can be described as a perception gap where people are not able to internalize, contextualize, and overview information but try to make the information fit with what they already believe (Byskov Petersen et al., 2020; Kahneman, 2013; Pickles et al., 2021). Our study participants used the fictitious case to indirectly relate information to their own situation. The case, thereby, functioned as a buffer between the participants and the information thus temporarily filling the perception gap. When participants had this medium to insert their reflections, worries, thoughts, or emotion, they were not immediately forced to relate the information directly to themselves. In this situation, the participants were more open to discuss screening harms that were discordant with their existing beliefs about the benefits of PSA screening. This might hint that this methodology is especially apposite when there are strong beliefs about the research topic. Future work could focus on improving this buffer that the case served as, to help participants transfer this improvement in knowledge directly to their own decision about screening.

Another study also found that when interviewed about experiences with cancer pathways, people actively distanced themselves from a distinct “worried type” (Damhus et al., 2022). This was similar to our findings where the participants proclaimed Flemming and Anne as worried types, while distancing themselves from them. Our suggested method might serve as a way to examine participants’ experiences with worry while shielding them of the apparent embarrassment of posing as the “worrying kind”.

Focus groups produce a special type of knowledge concerned with group dynamics and norms generated through group interaction. Therefore, focus groups research can complement health communication by revealing underlying ideas about specific health issues and the values tied to these ideas (Puchta & Potter, 2002). In our study, participants were able to express opinions discordant with group norms under the disguise of the fictitious case. Revealing unpopular opinions and underlying norms is crucial in research about sensitive topics such as prostate cancer and PSA screening. This knowledge might help improve communication about prostate cancer and PSA screening by addressing the norms tied to these topics.

Previous research has found that the use of cases in information material had a positive effect on prejudices due to the perspective-taking nature of the case presented in the material (Johnson et al., 2013). Another study suggested that people are more likely to trust information that is discordant with existing beliefs if this information is presented as a narrative (Slater & Rouner, 1996). Therefore, a fictitious case might accommodate potential prejudices and distrust in health communication.

Our findings suggest that participants did not “hold back” due to the sensitive nature of the subject. Therefore, adding a fictitious case may allow for greater depth to discussions in focus groups and therefore increase chances of a more meaningful analysis and interpretation to answer a research question relating to more sensitive topics.

### Strengths and limitations

A strength of this study was the theoretical foundation and the great variety of backgrounds among authors that allowed for diverse perspectives, reflections, and experiences (Shaffer et al., 2018).

We employed a fictitious case as a projective technique. In other words, we combined two approaches into one, which makes up the novelty of the proposed methodology. This use of a case as a projective technique can have similar effects to the use of vignettes. However, to our knowledge no studies have examined the intentional use of vignettes or cases as projective techniques. The effects of using a case as a projective technique might not be new but the intentional and reflective use of it is.

A limitation of this study, was that it only involved two small and homogenous focus groups with four men and their respective partners. However, we did not aim for generalization, rather to inspire future research on sensitive topics. Yet it means that the case may therefore function differently in other settings or in other groups. For example, people are more likely to self-disclose and share personal experiences in homogenous groups as participants are more likely to feel empowered and supported in situations, surrounded by peers (Farquhar, 1999). Therefore, the sense of safety to disclose emotions, might be partly caused by the group composition and exclusive to this setting. Future research might gain from this consideration in regard to their recruitment strategy in focus groups on sensitive topics.

### Implications for research

Because of the limited empirical data presented in this article, we suggest that future research test the usefulness of this approach before applying it on a larger scale. However, our preliminary work suggests that the projective method may enhance disclosure of worries, emotions, values, and experiences associated to cancer screening. The fictitious case was apposite for discussing prostate cancer and PSA testing as it shielded the participants from the embarrassment that could follow the sensitive nature of the topic.
